# Structure-Based Virtual Screening, Synthesis and Biological Evaluation of Potential FAK-FAT Domain Inhibitors for Treatment of Metastatic Cancer

**DOI:** 10.3390/molecules25153488

**Published:** 2020-07-31

**Authors:** Sahar B. Kandil, Samuel R. Jones, Sonia Smith, Stephen E. Hiscox, Andrew D. Westwell

**Affiliations:** School of Pharmacy and Pharmaceutical Sciences, Cardiff University, Wales CF10 3NB, UK; jonessr15@cardiff.ac.uk (S.R.J.); sonia.m.smith@hotmail.co.uk (S.S.); hiscoxse1@cardiff.ac.uk (S.E.H.); westwella@cardiff.ac.uk (A.D.W.)

**Keywords:** focal adhesion kinase (FAK), breast cancer (BC), pancreatic cancer, triple negative breast cancer (TNBC), chloropyramine (C4), rational drug design, virtual screening

## Abstract

Focal adhesion kinase (FAK) is a tyrosine kinase that is overexpressed and activated in several advanced-stage solid cancers. In cancer cells, FAK promotes the progression and metastasis of tumours. In this study, we used structure-based virtual screening to filter a library of more than 210K compounds against the focal adhesion targeting FAK-focal adhesion targeting (FAT) domain to identify 25 virtual hit compounds which were screened in the invasive breast cancer line (MDA-MB-231). Most notably, compound **I** showed low micromolar antiproliferative activity, as well as antimigratory activity. Moreover, examination in a model of triple negative breast cancer (TNBC), revealed that, despite not effecting FAK phosphorylation, compound **I** significantly impairs proliferation whilst impairing focal adhesion growth and turnover leading to reduced migration. Further optimisation and synthesis of analogues of the lead compound **I** using a four-step synthetic procedure was performed, and analogues were assessed for their antiproliferative activity against three breast cancer (MDA-MB-231, T47D, BT474) cell lines and one pancreatic cancer (MIAPaCa2) cell line. Compound **5f** was identified as a promising lead compound with IC_50_ values in the range of 4.59–5.28 μM in MDA-MB-231, T47D, BT474, and MIAPaCa2. Molecular modelling and pharmacokinetic studies provided more insight into the therapeutic features of this new series.

## 1. Introduction 

Focal adhesion kinase (FAK) was first described more than 20 years ago and represents a promising target for the treatment of several advanced-stage solid cancers, as well as mesothelioma and haematological malignancies. FAK is a multifunctional non-receptor tyrosine kinase whose overexpression and activation has been linked to tumour progression, survival, migration, invasion, epithelial-to-mesenchymal transition (EMT), and drug resistance through effects on the cancer cells as well as stromal cells of the tumour microenvironment [1,2]. FAK also exists at low levels in normal tissues or benign tumours [2]. The amplification of FAK in a number of solid cancers (e.g., colorectal, ovarian, breast, prostate, and sarcoma) has been previously documented [3,4,5,6,7,8]. FAK plays a pivotal role in protecting pancreatic ductal adenocarcinomas, through the development of an immunosuppressive and fibrotic microenvironment, and promoting immune evasion [9,10].

**Figure 1:**
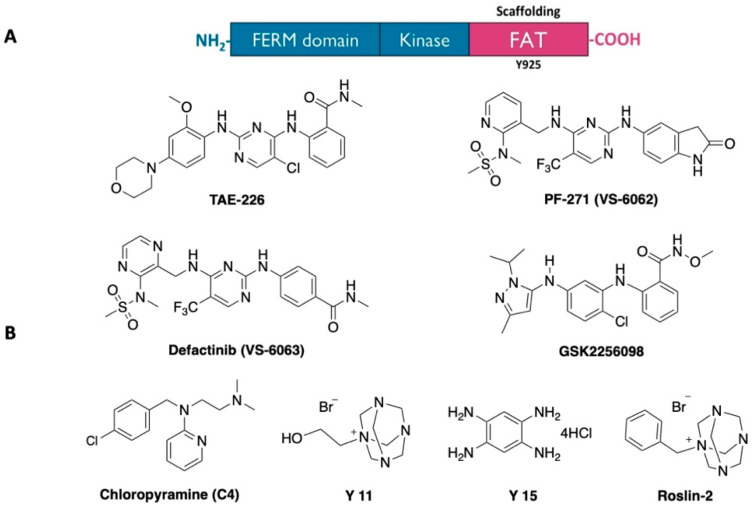
(**A**) Schematic representation of focal adhesion kinase (FAK); FERM is the N-terminal domain followed by the central kinase domain. The focal adhesion targeting domain (FAT) is the C-terminal part of the protein, showing key tyrosine phosphorylation site Y925. (**B**) Chemical structure of the kinase-dependent FAK inhibitors TAE-226, PF-271 (VS-6062), Defactinib (VS-6063), and GSK2256098; and the kinase-independent chloropyramine (C4), Y 15, Y 11, and Roslin-2 compounds.

FAK exerts its effects on cancer cells, as well as stromal cells of the tumour microenvironment, through both kinase-dependent and kinase-independent effects. In humans, FAK consists of the amino N-terminal region containing the FERM (4.1 protein-ezrin-radixin-moesin) domain, the central kinase domain, and the C-terminal focal adhesion targeting (FAT) domain (Figure 1). FAK possesses both kinase-dependent and kinase-independent (scaffolding) functions which control cell movement, invasion, survival, gene expression, and cancer stem cell self-renewal and is therefore an attractive target for anticancer therapy [11,12,13]. It is established that FAK phosphorylation and activation drives many tumour-related processes [2]. The therapeutic approach of FAK function inhibition in cancer cells can be subdivided into two main strategies [14].

The first strategy has been to develop ATP-competitive kinase inhibitors which are designed to bind residues surrounding the ATP-binding pocket of FAK around the tyrosine 397 (Y397) autophosphorylation site. As this pocket is similar in many different kinases, there is the potential for off-target effects stemming from a lack of kinase selectivity. For example, compound TAE-226 (Figure 1) which is a small ATP-competitive molecule, exhibits nanomolar activity, prevents cell invasion, reduces cell proliferation, increases apoptosis, and enhances docetaxel-mediated growth inhibition both in vitro and in vivo [15,16,17,18]. Nevertheless, the development of this molecule was later abandoned due to off-target effects. GlaxoSmithKline developed compound GSK2256098 (Figure 1) that displayed minor clinical responses in mesothelioma patients. When tested in combination with trametinib (MEK inhibitor), GSK2256098 did not demonstrate any improved efficacy compared to that observed with GSK2256098 monotherapy [19,20]. Additionally, compound PF-271 (VS-6062) (Figure 1) underwent Phase I clinical trial and was found to be tolerated with few adverse effects. However, PF-271 was discontinued because of its nonlinear pharmacokinetics profile [1]. Furthermore, Defactinib (VS-6063, PF-04554878) (Figure 1) is a late generation FAK inhibitor in phase I/II clinical trials and is tested as monotherapy as well as in combination with pembrolizumab or paclitaxel for patients with different types of solid tumours [21,22]. These studies suggest that targeting the FAK kinase activity may be promising especially when used in combination therapies. Notably, kinase selectivity is a well-known problem of many ATP binding site competitive inhibitors. Additionally, the ability of PYK2 (a FAK-related kinase) to take over certain FAK functions after FAK deletion has to be taken into account [14]. 

A second strategy towards targeting FAK is based on the inhibition of the key FAK kinase-independent (scaffolding) functions which are not blocked by FAK kinase inhibitors and are possibly enhanced by it. The scaffolding function effects help to explain why FAK kinase inhibition can lead to unpredictable therapeutic outcomes [23].

Moreover, several reports suggest that although FAK kinase activity is necessary for cell motility, it may be not essential for cell proliferation and survival. Additionally, to avoid selectivity problems typically found with competitive inhibitors of the ATP binding site, inhibiting the scaffolding function of FAK might be a useful alternative approach [14]. Targeting the FAT domain will potentially allow greater selectivity compared to the majority of other FAK inhibitors in clinical trials that target the kinase domain only. In this strategy, small molecules were identified via molecular modelling studies that may disrupt different scaffolding protein–protein interaction functions (PPI). These molecules include Y11, Y15, and Roslin (R2) plus chloropyramine hydrochloride (C4), a licenced H1 histamine receptor antagonist (Figure 1) [24,25,26]. It is proposed that C4 blocks FAK C-terminal focal adhesion targeting (FAT) domain interactions, inhibits cell proliferation in vitro, and reduces tumour growth in vivo within xenograft mouse models [27,28]. FAK inhibitors can also be used as chemotherapy sensitizers since they enhance the antitumour activity when used in combination with other cytotoxic chemotherapeutics; however, questions remain about target selectivity [17,21].

Increased FAK mRNA levels are found in several human malignancies. Large databases such as the Cancer Genome Atlas show that FAK mRNA levels are increased in invasive breast cancers (26%) [29], and that these increased levels are correlated with poor overall patient survival [30,31]. It is established that FAK has a crucial role and is considered to be an indicator of development, progression, and invasive potential of human breast cancer (BC). FAK shows upregulated expression, phosphorylation, and processing in BC tissue compared to the adjacent non-tumour tissue of the same patient. Upregulation of FAK was found to be increased in parallel with the advancement of cancer stages where FAK is upregulated both at protein and mRNA levels. Overexpression of FAK was frequently found in ER-positive and PR-positive BC, but not Her2/neu-negative breast cancer cases [32].

Moreover, the mRNA expression levels of the FAK encoding gene (PTK2), FAK phosphorylation as well as the focal adhesion points (FAs) are higher in triple negative breast cancer (TNBC), an aggressive BC type characterised by high metastatic potential. FAK inhibition prevented the oestrogen induced migration of invasive MDA-MB 231 cells [33]. Furthermore, FAK inhibition in a pancreatic cancer cell line (MiaPaCa2) resulted in a significant decrease in the number of colonies in a dose-dependent manner [34]. Additionally, treatment with FAK inhibitors resulted in decreased primary tumour size and fewer tumour-associated macrophages (TAMs), which are key contributors to tumour progression and inflammation, in a mouse model of pancreatic ductal adenocarcinoma [35]. Herein, we used a structure-based approach to design and synthesise potential FAK-FAT domain inhibitors.

## 2. Results and Discussion

### 2.1. Virtual Screening (Molecular Modelling)

The C-terminal focal adhesion targeting (FAT) domain of FAK is necessary for proper localisation of FAK to focal adhesions and subsequent activation. Phosphorylation of tyrosine 925 (Y925) in the FAT domain has been shown to promote tumour metastasis and invasion in vivo [36]. **C4** (chloropyramine hydrochloride) (Figure 1) is a clinically approved histamine receptor H1 antagonist and was identified in a previous study to inhibit FAK-FAT domain mediated signalling [27,28]. However, **C4** displays its anticancer activity at a remarkably high micromolar concentration (>100 μM). Our previous lead optimisation work on the chemical structure of chloropyramine (C4) led to the identification of a more active analogue against three human breast cancer lines (MDA-MB-231, BT474, and T47D) with an average antiproliferative activity (IC_50_) of approximately 23 μM [37].

**Figure 2:**
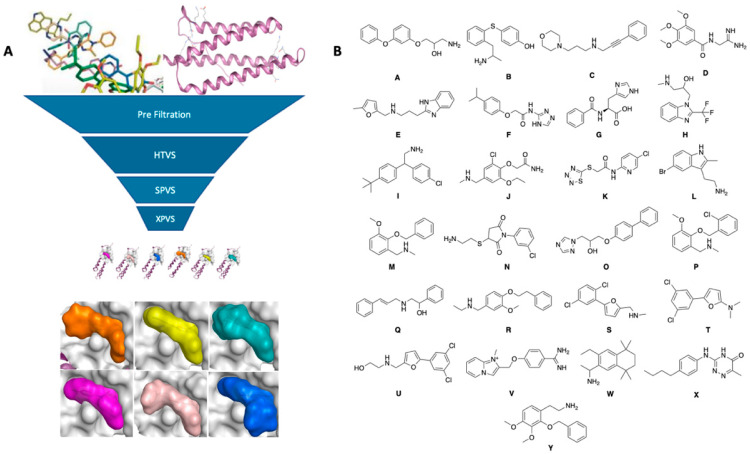
(**A**) Schematic representation of the virtual screening of SPECS virtual library against FAK-FAT domain. (**B**) Chemical structures of the top 25 preliminary FAK-FAT hits in the virtual screening experiment.

**Figure 3:**
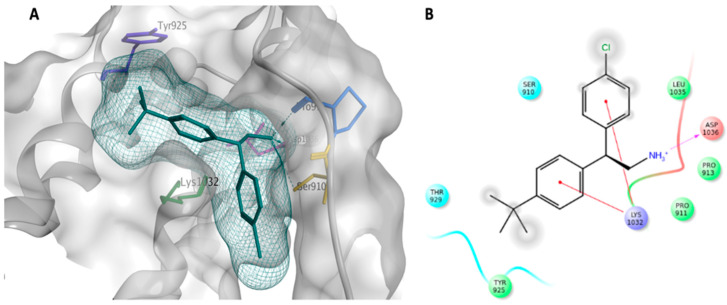
(**A**) The predicted binding mode of compound **I** (cyan sticks) inside the pocket of the FAK-FAT domain (grey) pocket, showing interaction with Asp 1036 (pink), Ser 910 (yellow), Pro 911 (blue), Lys 1032 (green), and Tyr 925 (purple); hydrogen bonds are indicated by the cyan dashed lines. (**B**) The detailed interactions between compound **I** and the FAK-FAT domain.

In this study, we aimed to use a structure-based drug design and virtual screening approach to identify a small molecule that can bind the FAK Tyr 925 phosphorylation site according to the workflow depicted in Figure 2A. Therefore, the crystal structure of the focal adhesion targeting (FAT) domain was obtained from the Protein Data Bank (PDB code 1K05) and prepared for docking [38]. The pocket used for the high throughout virtual screening was focused on the area surrounding the three amino acids: Asp 1036, Ser 910, and Lys 1032 which also encompasses the Tyr 925 residue (Figure 3).

Approximately 210,000 small molecules from the SPECS virtual library of compounds were imported, rendered, and minimised using a MMFF94x force field and run in a virtual screening simulations programme using Glide SP within Maestro software (Glide, version 9.5, Schrödinger; http://www.schrodinger.com). These compounds underwent four stages of filtration including prefiltration of the non-drug-like compounds in accordance with Lipinski’s rules and excluding chemically unstable and potentially toxic groups. This was followed by high throughput virtual screening (HTVS), standard precision virtual screening (SPVS) and extra precision virtual screening (XPVS). Each compound was positioned in the chosen pocket, which was made up of the area surrounding the three amino acids Asp 1036, Ser 910, and Lys 1032 within a 12 A° radius and scored for electrostatic and van der Waals interactions as implemented in the Schrodinger virtual screening package (version 9.5). This process resulted in the identification of twenty-five virtual hits (Figure 2B). The predicted binding modes to the FAK-FAT domain of selected hits are shown in Figure 2A. These 25 hits underwent preliminary in vitro profiling in the metastatic triple negative breast cancer (TNBC) cell line (MDA-MB-231) for antiproliferative and antimigratory properties. This revealed that compound **I** in particular exhibited low micromolar antiproliferative and antimigratory properties exceeding those of chloropyramine (**C4**) and thus was considered as a hit compound.

**Figure 4:**
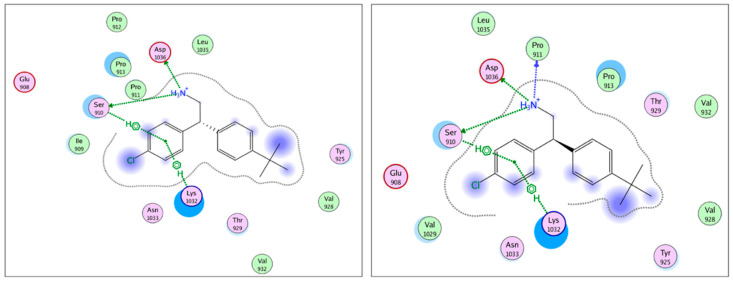
The predicted binding mode of the two enantiomers of compound **I** showing similar interactions with the FAK-FAT domain; hydrogen bond is indicated by the dashed lines.

According to the surface representation of the FAK-FAT domain, the predicted docking mode of compound **I** binds in a comparatively polar cavity surrounded by residues Ser 910, Thr 929, Pro 911, Pro 913, Lys 1032, and Asp 1036, as shown in Figure 3. Key interactions of compound **I** due to the presence of the free amine (NH_2_) group include a salt bridge with the side chain carboxyl group (COOH) of Asp 1036, two H-bonds with the backbone carbonyl (C=O) group of Pro 911, and the side chain hydroxy group (OH) of Ser 910. Additionally, hydrophobic interactions were noticed between the p-tert-butyl phenyl and p-chlorophenyl moieties and the surrounding hydrophobic pocket formed of residues Tyr 925, Val 928, Val 932, Pro 913, and Leu 1035 (Figure 3). Compound **I** possesses a chiral centre and both isomers are accommodated at the binding site and bind similarly with minimal difference in binding potential between enantiomers (Figure 4).

**Figure 5:**
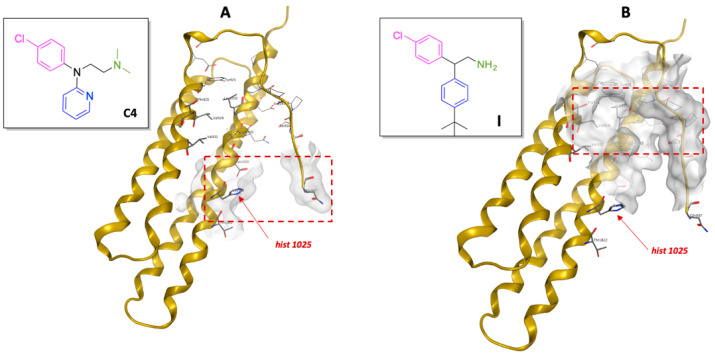
Comparison between the site selection (red dashed box) for the high throughout virtual screening of the drug-like FAK-FAT inhibitor; (**A**) previously identified chloropyramine (**C4**) centred around His 1025. (**B**) Our virtual screening hit compound **I** centred around the Asp 1036, Ser 910, and Lys 1032 pocket.

It is worth mentioning that the previous study published by Kurenova et al. [27] used the nuclear magnetic resonance analysis (NMR) of the FAT/VEGFR-3 peptide complex to localise the chemical shift of residue histidine 1025 on the FAT domain, to hypothesise that a small molecule binding to this site could disrupt the FAK-VEGFR-3 interaction (Figure 5A). On the other hand, our virtual screening study focused on the pocket near the catalytic tyrosine 925, which provides the chemical and geometric features appropriate for small molecule binding as indicated by the red box shown in Figure 5B. The grid generation module of the Schrodinger software was used to specify the pocket used to rank the potential ligands, based on the interactions with the target pocket (Figure 5B).

**Figure 6:**
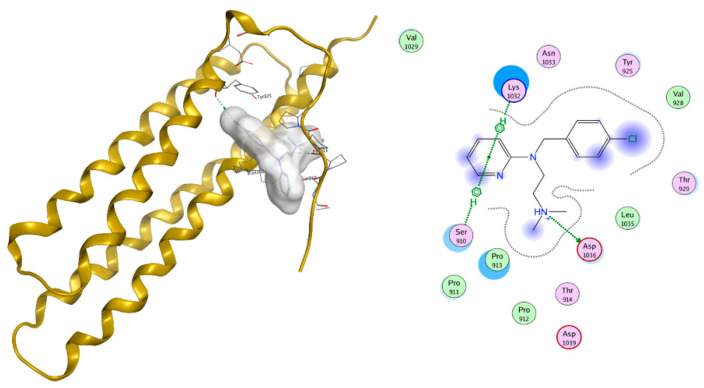
The predicted binding mode of chloropyramine (C4) showing detailed interactions with the pocket of the FAK-FAT domain; hydrogen bond is indicated by the dashed lines.

Interestingly, there appears to be some chemical structure similarity between compound **I** and **C4** (Figure 5). However, the significantly enhanced activity of compound **I** over **C4** appears to be a result of the improved interaction between the amino ethyl side chain of compound **I** and the COOH group of Asp 1036 (Figure 3), compared to **C4** (Figure 6).

### 2.2. Characterisation of the Cellular Effects of Compound **I**

#### 2.2.1. Compound **I** Inhibits TNBC Cell Proliferation and Migration

**Figure 7:**
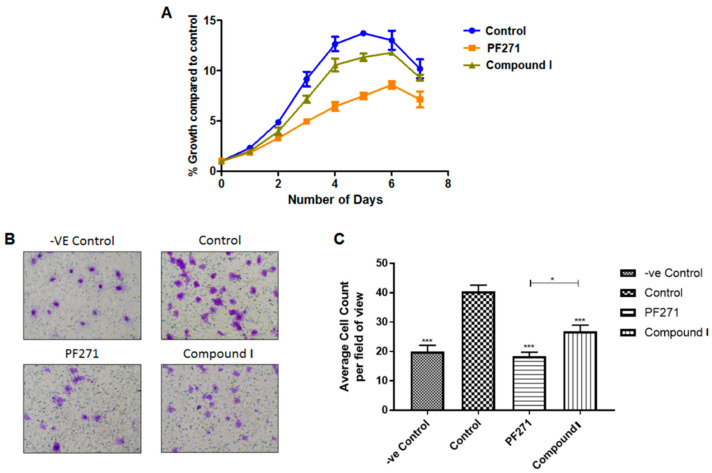
(**A**) Relative 7-day proliferation of MDA-MB-231 cells in vitro in the presence of 1 μM compound **I** or PF271 compared to vehicle-only control. (**B**) Representative images of Boyden chamber migration assays incubated with compound **I** or PF271, post 18 h serum-stimulated migration. (**C**) Quantified results of anti-migratory effects of compound **I** and PF271 from 18 h Boyden chamber migration assay. Negative (-ve) control represents non-serum stimulated cell migration. Data represent the average number of cells/membrane across duplicate wells and 3 replicates. Error bars show SEM: *p < 0.05; ***p < 0.001.

The effect of compound **I** on cell behaviour was explored in an in vitro model of TNBC, MDA-MB-231, using C4 and the FAK kinase inhibitor PF271 as control compounds. Initial Western blotting of FAK activity revealed no significant changes in the activity of either FAK^Y397^or FAK^Y925^, as well as no changes in the stability of total FAK protein following treatment with compound **I** (Figure S1; Supplementary Information). This was expected, given that this compound limits protein–protein scaffolding interaction rather than kinase activity and subsequent phosphorylation of key FAK residues. However, despite its inability to impair FAK phosphorylation, compound **I** could significantly reduce proliferation rates of MDA-MB-231 cells (Figure 7A). More relevant to FAK function, compound **I** also elicited a significant reduction in serum-stimulated migration in Boyden chamber assays (Figure 7B,C).

#### 2.2.2. Compound **I** Causes Changes in Cellular Morphology and Cellular Localisation of Active FAK 

**Figure 8:**
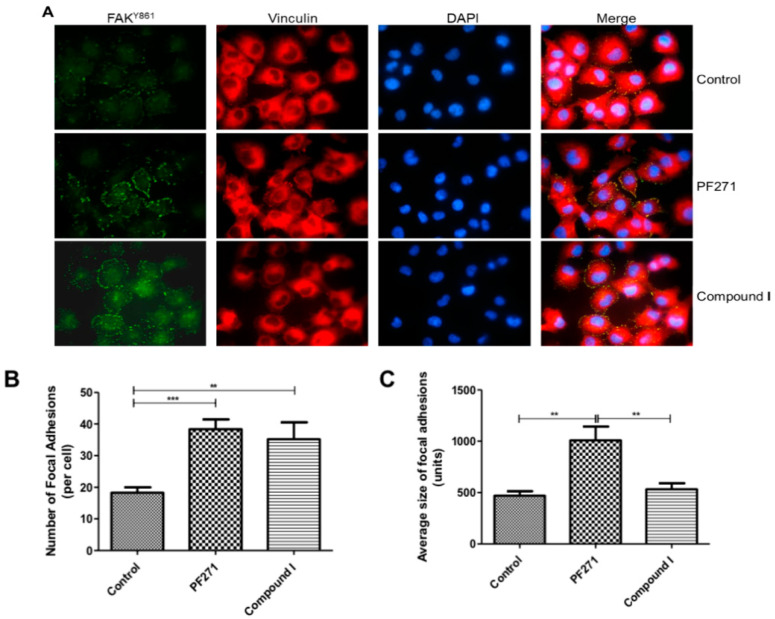
Exploration of subcellular dynamic changes in active FAK and FA-marker vinculin following compound **I** or PF271 treatment. (**A**) Images are representative of MDA-MB-231 cells following 1 h of serum stimulated migration in the presence of compound I, PF271, or a vehicle-only control. The generated images were subsequently used to quantify changes in the average number of focal adhesions/cell (**B**) and the average size of focal adhesions (**C**). All error bars represent SEM; n = 3. **p < 0.01; ***p < 0.001.

To validate that this change in migration was resulting from altered FAK functionality, we evaluated the changes in subcellular distribution of active FAK in response to compound **I**. Given its ability to bind and impair protein–protein interactions of the FAT-domain of FAK, we hypothesised that treatment could impair co-localisation and activity of FAK with focal adhesions. As such, we co-incubated MDA-MB-231 cells with a marker of active FAK (FAK^Y861^) with the well-established focal adhesion marker vinculin (Figure 8A). Both compound **I** and PF271 caused a significant alteration in FAK dynamics, with treatments leading to increased localisation of FAK to the cell periphery versus vehicle-only controls. However, compound **I** treated cells had significantly more active FAK displayed throughout the cytoplasm versus PF271 treated cells. These changes were also reflected in the dynamics of the focal adhesions, with a significant increase in focal adhesions being noted in treated cells (Figure 8B) implying impaired turnover of these regions. Interestingly, compound **I** had no effect on the size of focal adhesions, unlike PF271 (Figure 8C). Given the decrease in co-localisation with vinculin versus PF271, we hypothesised that compound **I** impairs migration through partial sequestering of active FAK to the cytoplasm, limiting its recruitment and activation of subsequent FAK-activated factors necessary for the growth and turnover of nascent focal adhesions.

### 2.3. Chemistry

The preliminary activity results suggest that compound **I** is an interesting starting point for further development, and that the aminoethyl group and the diaryl moiety are promising structural features. This prompted us to synthesise a series of compound **I** analogues to optimise the structure–activity profile. We investigated the impact of introducing various substituents to the diarylethylamine scaffold while keeping a bulky hydrophobic substituent at the para position of one of the phenyl groups.

**Scheme 1:**
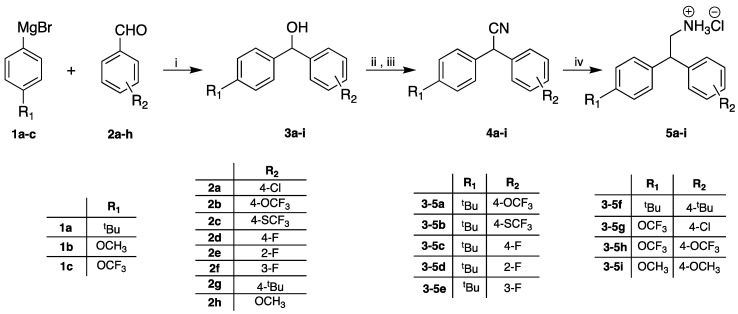
Synthesis of diphenylethylamine derivatives (**5a**–**i**). Reagents and conditions: (**i**) THF, rt, 24 h; (**ii**) SOCl_2_, CH_2_Cl_2_, rt, 3 h; (**iii**) TMSCN, TiCl_4_, CH_2_Cl_2_, rt, 24 h; (**iv**) LiAlH_4,_ Et_2_O, rt, 24 h, then 2 M HCl in anh. Et_2_O.

A four-step synthetic pathway was devised for the synthesis of compound **I** analogues. The hydrochloride salts of the diarylethylamine derivatives (**5a**–**i**) were prepared according to Scheme 1. The first step involved the reaction of the corresponding Grignard reagent (**1a**–**c**) and the respective aromatic aldehyde (**2a**–**h**) to prepare the diarylmethanol intermediates (**3a**–**i**). Pure products were obtained in good yields (47–77%). The conversion of the sterically congested alcohol group in **3a**–**i** into the corresponding nitrile group was achieved via two steps. Firstly, the generation of the chloride derivatives was achieved using thionyl chloride (SOCl_2_). Secondly, the reaction of the chloride derivatives with titanium tetrachloride (TiCl_4_) and trimethylsilylcyanide (TMSCN) successfully provided the nitrile analogues (**4a**–**i**). The reduction of the nitrile group into amine was achieved using lithium aluminium hydride (LiAlH_4_), followed by treatment with 2M hydrogen chloride in anhydrous diethyl ether to yield the target hydrochloride salts **5a**–**i** (Scheme 1). Column chromatography and/or recrystallisation were used to purify all compounds. Salt forms of the final compounds were prepared to give crystalline products and avoid the purification problems of the free amines. The confirmation of the structures of all the synthesised compounds was achieved using analytical and spectroscopic data (^1^H, ^13^C, ^19^F NMR, and mass spectrometry). The acquired data were in full accordance with the depicted structures. (Figures S2–S4; Supplementary Information).

### 2.4. Cell Viability Assay

**Figure 9:**
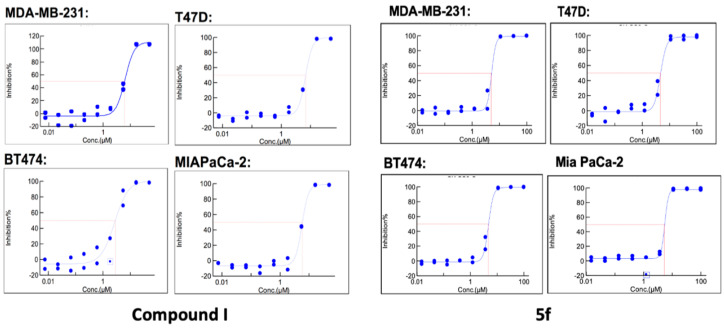
Antiproliferative activity of compounds **I** and **5f** across three human breast cancer cell lines (MDA-MB-231, BT474, and T47D) and one pancreatic cancer (Mia PaCa-2) cell line.

The antiproliferative activity of compound **I** as well as its newly synthesised analogues (**5a**–**i**) was evaluated in vitro against the three human breast cancer cell lines MDA-MB-231 (TNBC), T47D (ER^+^, HER^−^), and BT474 (ER^+^, HER^+^) as well as a human pancreatic cancer cell line MIAPaCa2. Chloropyramine (**C4**) was used as a positive control, and the results are shown in Table 1. The seeding of the corresponding cell lines started one day before incubation, which lasted for 72 h with the different concentrations of the tested compounds. CellTiter Glo reagent (25 μL) was added to the cell plate. The luminescence was analysed after 10 min using a PerkinElmer Envision instrument. The test compounds were used at ten different triplicate concentrations in half log increments up to 100 μM. The results showed that all compounds displayed better anti-proliferative activity than chloropyramine (**C4**). Notably, **C4** did not show any significant anti-proliferative activity up to a concentration of 100 μM across the three human breast (hBC) cell lines and one pancreatic cancer (hPC) cell line. Marginally, the best activity was associated with compound **5f**, which showed low micromolar activity in the three hBC cell lines (MDA-MB-231, IC_50_ = 5.06 μM), (BT474, IC_50_ = 4.59 μM), and (T47D, IC_50_ = 4.70 μM) and the pancreatic cancer line (MIAPaCa2, IC_50_ = 5.28 μM) (Figure 9).

**Figure 10:**
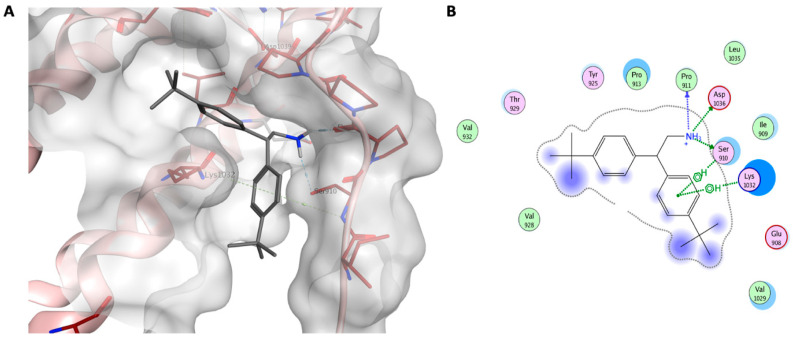
(**A**) The predicted binding mode of compound **5f** inside the pocket of the FAK-FAT domain showing hydrogen bonds as blue dashed lines. (**B**) The detailed interactions between compound **5f** and FAK-FAT domain.

Figure 10 illustrates the predicted docking mode of compound **5f** which offers the advantage of lacking the chiral centre present in compound **I**. Compound **5f** sits in the pocket surrounded by residues Ser 910, Thr 925, Thr 929, Pro 911, Pro 913, Lys 1032, and Asp 1036, as shown in Figure 5. Key interactions of compound **5f** due to the presence of the free amine (NH_2_) group include a salt bridge with the side chain carboxyl group (COOH) of Asp 1036, two H-bonds with the backbone carbonyl (C=O) group of Pro 911, and the side chain hydroxy group (OH) of Ser 910. In addition, hydrophobic interactions were noticed between the p-tert butyl phenyl moiety and the surrounding hydrophobic pocket formed of residues Tyr 925, Val 928, Val 932, Pro 913, and Leu 1035 (Figure 10).

### 2.5. Pharmacokinetic Studies

#### 2.5.1. In Vitro Aqueous Solubility

Furthermore, in vitro pharmacokinetic (PK) studies of the most active compounds were carried out. The in vitro aqueous solubility was evaluated using five different concentrations (1, 3, 10, 30, and 100 μM), after incubation at 37 °C for 2 h. Both the estimated precipitation range (lower and upper bound) and a mid-range value were measured. Nicardipine and pyrene were used as control compounds. Compound **I** and **5c** showed high aqueous solubility with an estimated precipitation range of 100 (lower bound) and >100 μM (upper bound). Compounds **5a** and **5h** showed moderate solubility of 30 μM (lower bound) and 100 μM (upper bound), and compounds **5b** and **5f** showed relatively reduced solubility comparable to pyrene (Table 2).

#### 2.5.2. Microsomal Metabolic Stability

The metabolic stability of compounds **I**, **5a**, **5c**, **5f**–**5h** was tested in human liver microsomes. All compounds were incubated for 45 min with pooled liver microsomes and the intrinsic clearance (CL_int_), which is the theoretical unrestricted maximum clearance of unbound drug without blood or plasma protein binding limitations. Half-life (t_1/2_) values were measured at five time points. Both CL_int_ as well as t_1/2_ values were compared to dextromethorphan and verapamil as control compounds (Table 3). Compound **5f** displayed a microsomal half-life (t_1/2_) = 148 min, which is higher than that of the drug controls—dextromethorphan (t_1/2_ = 49.2 min) and verapamil (t_1/2_ = 7.4 min)—and represents a reasonable starting point for the development of an anticancer therapy.

#### 2.5.3. Cardiotoxicity Assay

The hERG channel inhibition assay is a sensitive measurement to identify compounds exhibiting cardiotoxicity related to hERG channel inhibition. Compounds **I**, **5a**, **5f,** and **5i** were tested for hERG channel inhibition in the patch-clamp assay. The IC_50_ was calculated at 6 different concentration points (0, 0.008, 0.04, 0.2, 1, 5, and 25 µM). The results show that there was no in vitro cardiotoxicity observed with compound **5f** (IC_50_ = >25 µM) which reflects a satisfactory degree of safety for this compound (Table 4) [39]. (http://cyprotex.com). 

## 3. Materials and Methods

### 3.1. Molecular Modelling

The crystal structure of the focal adhesion targeting (FAT) domain was downloaded from the Protein Data Bank (PDB code 1K05) [38] and prepared for docking using the MOE (Molecular Operating Environment) protein preparation tools. The library of commercially available compounds was downloaded from the SPECS website [40] (www.specs.net) in sdf format and prepared using the conformational Search tool in MOE. The virtual screening simulations were performed using the Glide SP within Maestro software using the default settings (Glide, version 9.5, Schrödinger; http://www.schrodinger.com). The pocket used for the high throughout virtual screening was focused on the area surrounding the three amino acids: Asp 1036, Ser 910, and Lys 1032 which also encompasses the Tyr 925 residue within a 12A° radius. The virtual screening output database was saved as a mol2 file, and the visual inspection of the docking modes was performed in MOE. 

### 3.2. Cell Proliferation Assay

To assess proliferation, cells were seeded at 4.2 × 10^5^ cells/mL in 24-well plates. Following 24 h, the media were replaced with fresh RPMI + 5% FCS, in the presence or absence of treatments and a selection of wells counted (day 0 counts). Cells were subsequently counted each day up to 7 days to assess proliferation. Briefly, this entailed the removal of old media and the addition of trypsin/EDTA in order to lift cells before passing gently through a 25 G needle to achieve a single-cell suspension. The resulting solution was then added to isoton in a counting cup and the cell number was determined using a Coulter Multisizer III (Beckman Coulter Life Sciences, Indianopolis, IN, USA) Each well was counted twice with all conditions performed in triplicate.

### 3.3. Boyden Chamber Migration

Migration was assessed utilising 24-well, transmembrane permeable support plates (Corning Lifesciences, Corning, New York, NY, USA) with 6.5 mm microporous membrane (8 μm pore size) inserts, each coated with 10 μg/mL fibronectin in sterile PBS. Inserts were placed into wells containing RPMI + 5% FCS (± treatments), before cells in serum-free RPMI were seeded into the top portion of each insert at a density of 50,000 cells/mL. Cells were then incubated at 37 °C and 5% CO_2_ for 18 h. After this migratory period was completed, cells were fixed to the underside of the inserts in 3.7% PFA in PBS and stained with 0.5% crystal violet solution. Resulting inserts were then imaged using a standard light microscope.

### 3.4. Immunofluorescence

MDA-MB-231 TNBC cells were seeded at 700,000 cells/mL onto fibronectin coated coverslips and allowed to proliferate until 50% confluent, at which point they were treated with serum-free RPMI for 24 h. Following this, cells were incubated with RPMI+ 5% FCS for 1 h, before being washed briefly in sterile PBS, and fixed in 3.7% PFA for 15 min. The resulting fixed cells were subsequently permeabilised with 0.2% Triton-X100 in PBS for 8 min. This was proceeded by a 40 min block in 10% normal goat serum (in 1% BSA in PBS) prior to 30 min primary antibody incubation. Both FAK^Y861^ and vinculin antibodies were diluted to working concentration (1:100 and 1:200, respectively) in 1% BSA in PBS. Specific fluorophore-conjugated secondary antibodies were then applied (Alexafluor-488 and Alexfluor-594 diluted 1:1000 in 1% BSA in PBS), and resulting coverslips were briefly washed in PBS and mounted to glass slides using hard-set Vectashield mounting media plus DAPI (4′, 6-diamidino-2-phenylindole). Slides were viewed using a 63X oil immersion lens on a Leica DM IRE2 microscope (Leica Microsystems, Wetzlar, Germany).

### 3.5. General Procedure for the Preparation of Diaryl Alcohols (**3a**–**i**)

The diaryl methanol derivatives (**3a**–**i**) were prepared by the dropwise addition of the corresponding substituted phenyl magnesium bromide (Grignard reagent, **1a**–**c**) 0.5M solution in THF (5.7mmol) to a solution of the respective aldehyde (5.7mmol) (**2a**–**d**) in THF (20 mL) at 0 °C. The reaction mixture was stirred for 24 h from 0 °C to room temperature. The mixture was concentrated under vacuum then quenched by adding saturated aqueous NH_4_Cl (30 mL) and extracted with ethyl acetate (3 × 30 mL). The combined organic layers were washed with brine (20 mL) and water (30 mL), dried over anhydrous magnesium sulphate, filtered, and concentrated under vacuum. The crude residue was purified by column chromatography eluting with hexane-ethyl acetate gradually increasing from 100:0 to 90:10 v/v. Pure products were obtained in good yields (47%–77%).

*(4-tert-butylphenyl)(4-(trifluoromethoxy)phenyl)methanol* (**3a**), ^1^H NMR (CDCl_3_) δ 7.45 (d, J = 8.5 Hz, 2H, ArH), 7.41 (d, J = 8.5 Hz, 2H, ArH), 7.31 (d, J = 8.5 Hz, 2H, ArH), 7.21 (dd, J = 1, 9 Hz, 2H, ArH), 5.86 (d, J = 3 Hz, 1H, CH), 2.57 (d, J = 3.5 Hz, 1H, OH), 1.34 (s, 9H, 3 × CH_3_). ^19^F (CDCl_3_) δ −57.84. ^13^C NMR (CDCl_3_) δ 150.99 (ArC), 148.40 (ArC), 142.44 (ArC), 140.45 (ArC), 127.86 (ArCH), 126.32 (ArCH), 125.65 (ArCH), 120.90 (ArCH), 120.47 (^1^J_C-F_ = 255.5Hz, OCF_3_), 75.40 (CH), 34.58 (C(CH_3_)_3_), 31.33 (C(CH_3_)_3_), MS [ESI, *m*/*z*]: calcd for C_18_H_19_F_3_O_2_ [M + H-OH] 308.1388; found 308.1384.

*(4-tert-butylphenyl)(4-((trifluoromethyl)thio)phenyl)methanol* (**3b**), ^1^H NMR (CDCl_3_) δ 7.67 (d, J = 8.5 Hz, 2H, ArH), 7.48 (d, J = 8.5 Hz, 2H, ArH), 7.44 (d, J = 8.5 Hz, 2H, ArH), 7.30 (d, J = 8.5 Hz, 2H, ArH), 5.79 (d, J = 3 Hz, 1H, CH), 2.95 (d, J = 3.5 Hz, 1H, OH), 1.39 (s, 9H, (CH_3_)_3_). ^19^F (CDCl_3_) δ −42.65, ^13^C NMR (CDCl_3_) δ 151.09 (ArC), 146.88 (ArC), 140.20 (ArC), 136.35 (ArCH), 129.67 (q, ^1^J_C-F_ = 306.3 Hz, SCF_3_), 127.52 (ArCH), 126.49 (ArCH), 125.68 (ArCH), 75.49 (CH), 31.59 (C(CH_3_)_3_), 31.34 (CH_3_)_3_. MS [ESI, *m*/*z*]: calcd C_18_H_19_F_3_OS [M − H] 339.1031; found 339.1033.

*(4-tert-Butylphenyl)(4-fluorophenyl) methanol* (**3c**), ^1^H NMR (CDCl_3_) δ 7.44–7.36 (m, 4H, ArH), 7.34–7.28 (m, 2H, ArH), 7.05 (t, J = 8.5Hz, 2H ArH), 5.82 (s, 1H, CH), 2.50–2.25 (m, 1H, OH), 1.33 (m, 9H, (CH_3_)_3_. ^19^F (CDCl_3_) δ −115.24, ^13^C NMR (CDCl_3_) δ 162.15 (d, ^1^J_C-F_ = 243.8 Hz, ArC), 150.75 (ArC), 140.78 (ArC), 139.67 (ArC), 128.19 (d, ^3^J_C-F_ = 8.0 Hz, ArCH), 126.28 (ArCH), 125.53 (ArCH), 115.22 (d, ^2^J_C-F_ = 21.1 Hz, ArCH), 70.87 (CH), 31.50 C(CH_3_)_3_, 25.10 (CH_3_)_3_. MS [ESI, *m*/*z*]: calcd C_17_H_19_FO [M − H] 257.1342; found 257.1338 and [M−H_2_O + H] 241.1393; found 241.1390.

*(4-tert-butylphenyl)(2-fluorophenyl)methanol* (**3d**), ^1^H NMR (CDCl_3_) δ 7.57 (t, J = 7.5Hz, 1H, ArH), 7.39–7.35 (m, 4H, ArH), 7.29–7.26 (m, 2H, ArH), 7.19 (t, J = 7.5Hz, 1H, ArH), 6.16 (d, J = 4Hz, 1H, CH), 2.26 (d, J = 4.5Hz, 1H, OH), 1.33 (s, 9H, (CH_3_)_3_). ^19^F (CDCl_3_) δ −118.50, ^13^C NMR (CDCl_3_) δ 159.91 (d, ^1^J_C-F_ = 244.6 Hz, ArC), 150.71 (ArC), 139.79 (ArC), 131.04 (d, ^2^J_C-F_ = 12.1Hz, ArCH), 129.00 (d, ^2^J_C-F_ = 8.8 Hz, ArCH), 127.64 (d, ^2^J_C-F_ = 3.8 Hz, ArCH), 126.14 (ArC), 125.46 (ArC), 124.28 (d, ^1^J_C-F_ = 3.8 Hz, ArCH), 70.01 (CH), 34.53 (C(CH_3_)_3_), 31.33 (CH_3_)_3_. MS [ESI, *m*/*z*]: calcd C_17_H_19_FO [M − H] 257.1342; found 257.1341 and [M−H_2_O + H] 241.1; found 241.1.

*(4-tert-Butylphenyl)(3-fluorophenyl) methanol* (**3e**), ^1^H NMR (CDCl_3_) δ 7.40 (d, J = 8.5Hz, 2H, ArH), 7.35–7.29 (m, 3H, ArH), 7.21–7.14 (m, 2H, ArH), 7.97 (td, J = 2.5, 8.5Hz, 1H, ArH), 5.84 (d, J = 3Hz, 1H, CH), 2.21 (d, J = 3.5Hz, 1H, OH), 1.34 (s, 9H, (CH_3_)_3_). ^19^F (CDCl_3_) δ −112.92, ^13^C NMR (CDCl_3_) δ 162.94 (d, ^1^J_C-F_ = 244.5 Hz, ArC), 150.95 (ArC), 146.45 (d, ^2^J_C-F_ = 6.5 ArC), 140.40 (ArC), 129.87 (d, ^3^J_C-F_ = 8.1Hz, ArCH), 126.35 (ArCH),), 125.60 (ArCH), 122.00 (d, ^4^J_C-F_ = 3.1 Hz, ArCH), 114.23 (d, ^2^J_C-F_ = 20.9Hz, ArCH), 113.36 (d, ^2^J_C-F_ = 21.8 Hz, ArCH), 75.52 (d, ^4^J_C-F_ = 1.6 Hz CH), 34.56 (C(CH_3_)_3_), 31.32 (CH_3_)_3_. MS [ESI, *m*/*z*]: calcd C_17_H_19_FO [M − H] 257.1342; found 257.1341 and [M−H_2_O + H] 241.1; found 241.1.

*Bis(4-tert-butylphenyl)methanol* (**3f**), ^1^H NMR (CDCl_3_) δ 7.40–7.38 (m, 4H, ArH), 7.36–7.33 (m, 4H, ArH), 5.84 (d, J = 3Hz, OH), 2.16 (d, J = 3.5 Hz, CH), 1.34 (s, 2(CH_3_)_3_). ^13^C NMR (CDCl_3_) δ 150.40 (ArC), 141.00 (ArC), 126.25 (ArCH), 125.39 (ArCH), 79.95 (CH), 34.52 C(CH_3_)_3_, 31.36 (CH_3_)_3_. MS [ESI, *m*/*z*]: calcd for C_21_H_28_O [M − H] 295.2062; found 295.2061 and [M−H_2_O + H] 279.2113; found 279.2115.

*(4-Chlorophenyl)(4-trifluoromethoxyphenyl)methanol* (**3g**), ^1^H NMR (CDCl_3_) δ 7.38 (d, J = 8 Hz, 2H, ArH), 7.35 (d, J = 9 Hz, 2H, ArH), 7.30 (d, J = 8.5 Hz, 2H, ArH), 7.21 (dd, J = 1, 8.5 Hz, 2H, ArH), 5.80 (d, J = 3 Hz, 1H, CH), 2.57 (d, J = 3 Hz, 1H, OH). ^19^F (CDCl_3_) δ −57.85, ^13^C NMR (CDCl_3_) δ 148.69 (ArC), 141.99 (ArC), 141.77 (ArC), 133.69 (ArC), 128.81 (ArCH), 128.81 (ArCH), 127.91 (ArCH), 127.86 (ArCH), 121.07 (ArCH), 120.44 (^1^J_C-F_ = 255.5Hz, OCF_3_), 74.90 (CH). MS [ESI, *m*/*z*]: calcd for C_14_H_10_ClF_3_O_2_ [M + H-H_2_O] 285.0294; found 285.0298.

*Bis(4-trifluoromethoxyphenyl)methanol* (**3h**), ^1^H NMR (CDCl_3_) δ 7.41 (dtd, J = 8.5, 3, 1 Hz, 4H, ArH), 7.23 (dd, J = 9, 1 Hz, 4H, ArH), 5.86 (d, J = 3 Hz, 1H, CH), 2.50 (d, J = 3.5 Hz, 1H, OH). ^19^F (CDCl_3_) δ −57.91, ^13^C NMR (CDCl_3_) δ 148.70 (ArC), 141.87 (ArC), 127.93 (ArCH), 121.11 (ArCH), 120.45 (q, ^1^J_C-F_ = 255.6 Hz, OCF_3_), 74.82 (CH). MS [ESI, *m*/*z*]: calcd C_15_H_10_F_6_O_3_ [M − H] 351.0456; found 351.0456.

### 3.6. General Procedure for Formation of Diaryl Nitrile (**4a**–**i**)

A solution of thionyl chloride (10.80 mmol) and diaryl alcohol (**3a**–**i**, 7.20 mmol) in dichloromethane (2 mL) was stirred at room temperature for 2 h. The reaction was concentrated under reduced pressure to give the diaryl chloride. To a solution of the diaryl chloride (7.20 mmol) in dichloromethane (33.12 mL), trimethylsilyl cyanide (7.20 mmol) and titanium tetrachloride (7.20 mL, 1 M solution in dichloromethane) were added. After stirring under argon at room temperature for 2 h, the reaction was quenched with methanol (13.90 mL) and water (41.62 mL) and diluted with dichloromethane (104 mL). The organic layer was washed with saturated, aqueous sodium bicarbonate (68.25 mL) and water (68.25 mL), dried over MgSO_4_, filtered, and concentrated under reduced pressure to give the crude product.

*2-(4-tert-butylphenyl)-2-(4-trifluoromethoxyphenyl)acetonitrile (***4a**), ^1^H NMR (CDCl_3_) δ 7.48–7.41 (m, 4H, ArH), 7.31 (d, J = 8 Hz, 2H, ArH), 7.26 (d, J = 8 Hz, 2H, ArH), 5.18 (s, 1H, CH), 1.36 (s, 9H, 3 × CH_3_). ^19^F (CDCl_3_) δ −57.87. ^13^C NMR (CDCl_3_) δ 151.68 (ArC), 148.98 (ArC), 134.79 (ArC), 132.26 (ArC), 129.28 (ArCH), 127.39 (ArCH), 126.34 (ArCH), 121.58 (ArCH), 120.41 (^1^J_C-F_ = 256.4Hz, OCF_3_), 119.46 (CN), 41.58 (CH), 34.63 (C(CH_3_)_3_), 31.24 (C(CH_3_)_3_). MS [ESI, *m*/*z*]: calcd for C_19_H_18_F_3_NO [M + Na], 356.1233; found, 356.1236.

*2-(4-tert-butylphenyl)-2-(4-(trifluoromethyl)thiophenyl)acetonitrile* (**4b**), ^1^H NMR (CDCl_3_) δ 7.69 (d, J = 8 Hz, 2H, ArH), 7.47–7.42 (m, 4H, ArH), 7.30–7.27 (m, 2H, ArH), 5.17 (s, 1H, CH), 1.34 (s, 9H, (CH_3_)_3_). ^19^F (CDCl_3_) δ -42.45, ^13^C NMR (CDCl_3_) δ 151.81 (ArC), 139.04 (ArC), 136.89 (ArCH), 131.88 (ArC), 129.40 (q, ^1^J_C-F_ = 306 Hz, SCF_3_), 128.83 (ArCH), 127.43 (ArCH), 126.37 (ArCH), 124.61 (q, ^3^J_C-F_ = 2.3 Hz, ArC), 119.13 (CN), 41.93 (CH), 34.64 (C(CH_3_)_3_), 31.23 (CH_3_)_3_. MS [ESI, *m*/*z*]: calcd C_19_H_18_F_3_NS [M + H] 350.1190; found 350.1185, [M + H − HCN] 323.1081; found 323.1082.

*2-(4-tert-butylphenyl)-2-(4-fluorophenyl)acetonitrile* (**4c**), ^1^H NMR (CDCl_3_) δ 7.46 (d, J = 8.5Hz, 2H, ArH), 7.39 (dd, J = 5, 8Hz, 2H, ArH), 7.36 (d, 8.5Hz, 2H ArH), 7.10 (t, J = 9Hz, 2H ArH), 5.17 (s, 1H CH), 1.38–1.37 (m, 9H, (CH_3_)_3_). ^19^F (CDCl_3_) δ -113.86. ^13^C NMR (CDCl_3_) δ 162.45 (d, ^1^J_C-F_ = 246.3 Hz, ArCH), 151.492 (ArC), 132.82 (ArC), 132.14 (ArC), 129.55 (d, ^3^J_C-F_ = 7.5 Hz, ArCH), 127.40 (ArCH), 126.29 (ArCH), 116.06 (CN), 116.15 (d, ^2^J_C-F_ = 21.3 Hz, ArCH), 41.46 (CH), 34.63 (C(CH_3_)_3_), 31.31 (CH_3_)_3_. MS [ESI, *m*/*z*]: calcd C_18_H_18_FN [M + NH_4_] 285.1767; found 285.1766.

*2-(4-tert-Butylphenyl)-2-(2-fluorophenyl)acetonitrile* (**4d**), ^1^H NMR (CDCl_3_) δ 7.38 (t, J = 7.5Hz, 1H, ArH), 7.30 (d, J = 7Hz, 2H ArH), 7.26–7.22 (m, 3H, ArH), 7.10 (t, J = 7.5Hz, 1H, ArH), 7.01 (t, J = 9Hz, 1H, ArH), 5.34 (s, 1H CH), 1.22 (s, 9H, (CH_3_)_3_). ^19^F (CDCl_3_) δ -117.28. ^13^C NMR (CDCl_3_) δ 159.70 (d, ^1^J_C-F_ = 246.3 Hz, ArC), 151.43 (ArC), 131.65 (ArC), 130.26 (d, ^3^J_C-F_ = 7.5 Hz, ArCH), 129.31 (d, ^4^J_C-F_ = 2.8 Hz, ArCH), 127.17 (ArCH), 126.15 (ArCH), 124.92 (d, ^3^J_C-F_ = 3.8 Hz, ArCH), 123.66 (d, ^2^J_C-F_ = 14.1 Hz, ArC), 119.05 (CN), 116.25 (d, ^2^J_C-F_ = 21.4 Hz, ArCH), 35.66 (d, ^3^J_C-F_ = 3.8 Hz, CH), 34.59 C(CH_3_)_3_, 31.25 (CH_3_)_3_. MS [ESI, *m*/*z*]: calcd C_18_H_18_FN [M + NH_4_] 285.1767; found 285.1765.

*2-(4-tert-butylphenyl)-2-(3-fluorophenyl)acetonitrile* (**4e**), ^1^H NMR (CDCl_3_) δ 7.43 (d, J = 8.5Hz, 1H, ArH), 7.41–7.35 (m, 1H, ArH), 7.29 (d, J = 8, 2H, ArH), 7.20 (d, J = 7.5Hz, 1H, ArH), 7.11- 7.02 (m, 2H, ArH), 5.13 (s, 1H CH), 1.34 (s, 9H, (CH_3_)_3_). ^19^F (CDCl_3_) δ -111.36. ^13^C NMR (CDCl_3_) δ 162.99 (d, ^1^J_C-F_ = 246.4 Hz, ArC), 151.65 (ArC), 138.37 (d, ^3^J_C-F_ = 7.3 Hz, ArC), 132.15 (ArC), 130.73 (d, ^3^J_C-F_ = 8.3 Hz, ArCH), 127.38 (ArCH), 126.28 (ArCH), 123.41 (d, ^4^J_C-F_ = 2.8 Hz, ArCH), 119.31 (CN), 115.28 (d, ^2^J_C-F_ = 21.0 Hz, ArCH), 114.99 (d, ^2^J_C-F_ = 22.8 Hz, ArC), 41.89 (d, ^4^J_C-F_ = 1.9 Hz, CH), 34.62 C(CH_3_)_3_, 31.24 (CH_3_)_3_. MS [ESI, *m*/*z*]: calcd C_18_H_18_FN [M + NH_4_] 268.1501; found 268.1494.

*2,2-Bis(4-tert-butylphenyl)acetonitrile* (**4f**), ^1^H NMR (CDCl_3_) δ 7.42–7.40 (m, 4H, ArH), 7.31–7.29 (m, 4H, ArH), 5.11 (s, 1H, CH), 1.33 (s, 9H, 2(CH_3_)_3_). ^13^C NMR (CDCl_3_) δ 151.20 (ArC), 133.03 (ArC), 127.39 (ArCH), 126.08 (ArCH), 120.04 (CN), 41.82 (CH), 34.57 C(CH_3_)_3_, 31.27 (CH_3_)_3_. MS [ESI, *m*/*z*]: calcd C_22_H_27_N [M + NH_4_] 323.2487; found 323.2487.

*2-(4-Chlorophenyl)-2-(4-trifluoromethoxyphenyl)acetonitrile* (**4g**), ^1^H NMR (CDCl_3_) δ 7.42–7.37 (m, 4H, ArH), 7.31 (d, J = 8 Hz, 2H, ArH), 7.26 (dd, J = 1, 9 Hz, 2H, ArH), 5.16 (s, 1H, CH). ^19^F (CDCl_3_) δ -57.90.^13^C NMR (CDCl_3_) δ 149.19 (ArC), 134.73 (ArC), 134.04 (ArC), 133.78 (ArC), 129.59 (ArCH), 129.23 (ArCH), 129.06 (ArCH), 121.75 (ArCH), 120.35 (^1^J_C-F_ = 256.5Hz, OCF_3_), 118.83 (CN), 41.37 (CH). MS [ESI, *m*/*z*]: calcd for C_15_H_9_ClF_3_NO [M] 311.0325; found 311.0327.

*2,2-Bis(4-trifluoromethoxyphenyl)acetonitrile* (**4h**), ^1^H NMR (CDCl_3_) δ 7.41 (d, J = 8.5 Hz, 4H, ArH), 7.28 (d, J = 9 Hz, 4H, ArH), 5.21 (s, 1H, CH). ^19^F (CDCl_3_) δ -57.97, ^13^C NMR (CDCl_3_) δ 149.26 (ArC), 133.93 (ArC), 129.27 (ArCH), 121.76 (ArCH), 120.36 (q, ^1^J_C-F_ = 256.4 Hz, OCF_3_), 118.83 (CN), 41.30 (CH). MS [ESI, *m*/*z*]: calcd C_16_H_9_F_6_NO_2_ [M] 361.0537; found 361.0531, [M + H] 362.0616; found 362.0595, [M + H-HCN] 335.0507; found 335.0530.

*2,2-Bis(4-methoxyphenyl)acetonitrile* (**4i**), ^1^H NMR (CDCl_3_) δ 7.27 (d, J = 9 Hz, 4H, ArH), 6.91 (d, J = 8.5 Hz, 4H, ArH), 5.08 (s, 1H, CH), 3.83 (s, 6H, 2xCH_3_). ^13^C NMR (CDCl_3_) δ 159.40 (ArC), 128.82 (ArCH), 128.30 (ArC), 120.13 (CN), 114.50 (ArCH), 55.36 (2xCH_3_), 41.07 (CH). MS [ESI, *m*/*z*]: calcd for C_16_H_15_NO_2_ [M − H] 252.1019; found 252.1021.

### 3.7. General Procedure for the Reduction of Nitrile to Amine Hydrochloride (**5a**–**i**)

To a suspension of lithium aluminum hydride (2.35 mmol) in anhydrous diethylether (20 mL) at 0 °C, a solution of nitrile (**4a**–**i**, 0.78 mmol) in diethylether (10 mL) was added dropwise. After stirring under nitrogen for 24 h, the reaction was quenched with water (10 mL). The reaction mixture was extracted with ethyl acetate (3 × 50mL). The combined organic layer was dried over MgSO_4_, filtered, and concentrated under reduced pressure to give the crude product. The crude mixture was treated with a 2 M anhydrous HCl solution in diethyl ether (5–10 mL), and the resulting amine hydrochloride salts were washed with diethyl ether. If the amine hydrochloride salts did not form a precipitate, the diethyl ether solution was concentrated under reduced pressure and rinsed with diethyl ether to give the hydrochloride salts.

*2-(4-tert-butylphenyl)-2-(4-trifluoromethoxyphenyl)ethan-1-aminium hydrochloride* (**5a**), ^1^H NMR (CDCl_3_) δ 8.12 (bs, 3H, ^+^NH_3_), 7.35 (d, J = 8 Hz, 2H, ArH), 7.31 (d, J = 8.5 Hz, 2H, ArH), 7.17 (d, J = 8.5, 2H, ArH), 7.14 (d, J = 8.5, 2H, ArH), 4.44 (t, J = 8 Hz, 1H, CH), 3.57 -3.42 (m, 2H, CH_2_), 1.29 (s, 9H, 3xCH_3_). ^19^F (CDCl_3_) δ −57.81. ^13^C NMR (CDCl_3_) δ 150.89 (ArC), 148.50 (ArC), 138.30 (ArC), 135.90 (ArC), 129.53 (ArCH), 127.40 (ArCH), 126.19 (ArCH), 121.49 (ArCH), 120.39 (^1^J_C-F_ = 255.6Hz, OCF_3_), 47.57 (CH), 43.78 (CH_2_), 34.49 (C(CH_3_)_3_), 31.23 (C(CH_3_)_3_). MS [ESI, *m*/*z*]: calcd for C_19_H_23_ClF_3_NO [M + H] 338.1727; found 338.1726.

*2-(4-tert-butylphenyl)-2-(4-(trifluoromethyl)thiophenyl)ethan-1-aminium chloride* (**5b**), ^19^F (CDCl_3_) δ −42.45, ^1^H NMR (CDCl_3_) δ 8.05 (bs, 3H, ^+^NH_3_), 7.43–7.32 (m, 4H, ArH), 7.27–7.10 (m, 4H, ArH), 4.49 (t, J = 8Hz, 1H, CH), 3.60–3.30 (m, 2H, CH_2_), 1.31 (s, 9H, (CH_3_)_3_). ^13^C NMR (CDCl_3_) δ 151.49 (ArC), 139.63 (ArC), 136.98 (ArCH), 130.78 (ArC), 128.24 (q, ^1^J_C-F_ = 296 Hz, SCF_3_), 128.01 (ArCH), 127.41 (ArCH), 126.38 (ArCH), 42.15 (CH), 34.43 C(CH_3_)_3_, 31.30 C(CH_3_)_3_. MS [ESI, *m*/*z*]: calcd C_19_H_23_ClF_3_NS [M + H]^+^354.1498; found 354.1501.

*2-(4-tert-butylphenyl)-2-(4-fluorophenyl)ethan-1-aminium chloride* (**5c**), ^19^F (CDCl_3_) δ −114.15, ^1^H NMR (CDCl_3_) δ 8.07 (bs, 3H, ^+^NH_3_), 7.36–7.31 (m, 4H, ArH), 7.03–6.94 (m, 4H, ArH), 4.42 (t, J = 8Hz, 1H, CH), 3.51–3.50 (m, 2H, CH_2_), 1.29 (s, 9H, (CH_3_)_3_). ^13^C NMR (CDCl_3_) δ 161.99 (d, ^1^J_C-F_ = 245.0 Hz, ArCH), 150.48 (ArC), 136.73 (ArC), 135.58 (ArC), 129.78 (d, ^3^J_C-F_ = 7.9 Hz, ArCH), 127.40 (ArCH), 126.03 (ArCH), 125.57 (d, ^2^J_C-F_ = 20.0 Hz, ArCH), 34.45 (CH), 34.46 (CH_2_), 31.25 C(CH_3_)_3_, 29.71 (CH_3_). MS [ESI, *m*/*z*]: calcd C_18_H_23_ClFN [M-Cl]^+^ 272.1809; found 272.1808.

*2-(4-tert-butylphenyl)-2-(2-fluorophenyl)ethan-1-aminium chloride* (**5d**), ^1^H NMR (CDCl_3_) δ 8.10 (bs, 3H, ^+^NH_3_), 7.35–7.29 (m, 4H, ArH), 7.22–7.19 (m, 4H, ArH), 3.94 (t, J = 8Hz, 1H, CH), 3.62–3.57 (m, 1H, CH_2_), 3.52–3.48 (m, 1H, 1.32, CH_2_), 1.29 (s, 9H, (CH_3_)_3_). ^19^F (CDCl_3_) δ -115.13. ^13^C NMR (CDCl_3_) δ 149.70 (d, ^1^J_C-F_ = 256.1 Hz, ArC), 143.94 (ArC), 135.51 (ArC), 130.18 (d, ^3^J_C-F_ = 8.5 Hz, ArCH), 129.28 (d, ^4^J_C-F_ = 3.9 Hz, ArCH), 127.54 (ArCH), 126.00 (ArCH), 122.95 (d, ^3^J_C-F_ = 6.1 Hz, ArCH), 118.30 (d, ^2^J_C-F_ = 12.5 Hz, ArC), 116.32 (d, ^2^J_C-F_ = 22.5 Hz, ArCH), 42.82 (CH_2_), 31.25 (d, ^3^J_C-F_ = 3.4 Hz, CH), 30.56 (C(CH_3_)), 30.03 (CH_3_). C_18_H_23_ClFN [M-Cl]^+^, 272.1809; found 272.1806.

*2-(4-tert-butylphenyl)-2-(3-fluorophenyl)ethan-1-aminium chloride* (**5e**), ^1^H NMR (CDCl_3_) δ 8.15–7.81 (m, 3H, ^+^NH_3_), 7.38–7.32 (m, 4H, ArH), 7.26–7.20 (m, 2H, ArH), 7.19–7.16 (m, 2H, ArH), 4.48–4.40 (m, 1H, CH), 3.60–3.40 (m, 2H, CH_2_), 1.31–1.27 (m, 9H, (CH_3_)_3_). ^19^F (CDCl_3_) δ -111.70. ^13^C NMR (CDCl_3_) δ 163.06 (d, ^1^J_C-F_ = 245.3 Hz, ArC), 143.94 (ArC), 135.51 (ArC), 130.18 (d, ^3^J_C-F_ = 8.5 Hz, ArCH), 129.28 (d, ^4^J_C-F_ = 3.9 Hz, ArCH), 127.54 (ArCH), 126.00 (ArCH), 122.95 (d, ^3^J_C-F_ = 6.1 Hz, ArCH), 118.30 (d, ^2^J_C-F_ = 12.5 Hz, ArC), 116.32 (d, ^2^J_C-F_ = 22.5 Hz, ArCH), 43.71 (CH_2_), 47.79 (CH), 34.49 (C(CH_3_)_3_), 31.24 ((CH_3_)_3_). C_18_H_23_ClFN [M-Cl]^+^, 272.1809; found 272.1808.

*2,2-Bis(4-tert-butylphenyl)ethan-1-aminium chloride* (**5f**), ^1^H NMR (CDCl_3_) δ 8.08 (bs, 2H, NH_2_), 7.33 (d, J = 8Hz, 4H, ArH), 7.21 (d, J = 8Hz, 4H, ArH), 3.94 (t, J = 8Hz, 1H, CH), 3.52 (bs, 2H, CH_2_), 1.29 (s, 9H, 2(CH_3_)_3_). ^13^C NMR (CDCl_3_) δ 150.41 (ArC), 136.55 (ArC), 127.57 (ArCH), 126.05 (ArCH), 47.65 (CH), 44.08 (CH_2_), 34.46 C(CH_3_)_3_, 31.30 (CH_3_)_3_. MS [ESI, *m*/*z*]: calcd C_22_H_32_ClN [M + H] 310.2529; found 310.2529.

*2-(4-chlorophenyl)-2-(4-trifluoromethoxyphenyl)ethan-1-aminium chloride* (**5g**), ^1^H NMR (CDCl_3_) δ 8.13 (bs, 3H, ^+^NH_3_), 7.32 (d, J = 8 Hz, 2H, ArH), 7.27 (d, J = 8 Hz, 2H, ArH), 7.23–7.16 (m, 4H, ArH), 4.43 (t, J = 8 Hz, 1H, CH), 3.44 (bs, 2H, CH_2_). ^19^F (CDCl_3_) δ -57.85, ^13^C NMR (CDCl_3_) δ 148.69 (ArC), 137.72 (ArC), 137.15 (ArC), 133.88 (ArC), 129.44 (ArCH), 129.40 (ArCH), 129.31 (ArCH), 121.64 (ArCH), 120.37 (^1^J_C-F_ = 212.5Hz, OCF_3_), 47.48 (CH), 43.31 (CH_2_). MS [ESI, *m*/*z*]: calcd for C_15_H_14_Cl_2_F_3_NO [M + H] 316.0711; found 316.0712.

*2,2-Bis(4-trifluoromethoxyphenyl)ethan-1-aminium chloride* (**5h**), ^19^F (CDCl_3_) δ −57.84, ^1^H NMR (CDCl_3_) δ 8.15 (bs, 3H, ^+^NH_3_), 7.42–7.33 (m, 4H, ArH), 7.12–7.08 (m, 4H, ArH), 4.26–4.20 (m, 1H, CH), 3.35–3.28 (m, 2H, CH_2_). ^13^C NMR (CDCl_3_) δ 148.27 (ArC), 142.90 (ArC), 128.88 (ArCH), 121.34 (q, ^1^J_C-F_=252.3 Hz, OCF_3_), 120.96 (ArCH), 52.25 (CH), 50.71 (CH_2_). MS [ESI, *m*/*z*]: calcd C_16_H_14_ClF_6_NO_2_ [M + H]^+^ 366.0923; found 366.0924.

*2,2-Bis(4-methoxyphenyl)ethan-1-aminium chloride* (**5i**), ^1^H NMR (CDCl_3_) δ 8.04 (bs, 3H, ^+^NH_3_), 7.17 (d, J = 7.5 Hz, 4H, ArH), 6.85 (d, J = 7.5 Hz, 4H, ArH), 4.38 (t, J = 8.5 Hz, 1H, CH), 3.76 (s, 6H, 2OCH_3_), 3.48 (bs, 2H, CH_2_). ^13^C NMR (CDCl_3_) δ 158.59 (ArC), 133.15 (ArC), 128.95 (ArCH), 114.31 (ArCH), 55.25 (2xCH_3_), 53.27 (CH), 45.23 (CH_2_). MS [ESI, *m*/*z*]: calcd for C_16_H_20_ClNO_2_ [M + H] ^+^ 258.1489; found 258.1486.

### 3.8. In Vitro Aqueous Solubility, Metabolic Stability and Cardiotoxicity Studies

All in vitro biological evaluations were performed by Cyprotex, according to their internal procedures. The detailed protocols procedure can be found online.

#### 3.8.1. Turbidimetric Aqueous Solubility

The test compound was diluted in buffer to give a range of concentrations (1, 3, 10, 30, and 100 μM; final DMSO concentration 1%) and incubated at 37 °C for 2 hr. Absorbance was measured at a wavelength of 620 nm and the solubility was estimated from the concentration of the test compound that produces an increase in absorbance above the vehicle control (i.e., 1% DMSO in buffer). 

##### Experimental Procedure

The test compound (10 mM in DMSO) was serially diluted to give solutions of 0.1, 0.3, 1, and 3 mM in DMSO. Each test compound concentration was then further diluted 1 to 100 in buffer (typically 0.01 M phosphate buffered saline pH 7.4) so that the final DMSO concentration was 1%, and the final test compound concentrations were 1, 3, 10, 30, and 100 μM. The experiment was performed at 37 °C and each concentration was incubated in 7 replicate wells. The plates were incubated for 2 h at 37 °C before the absorbance was measured at 620 nm. Nicardipine and pyrene were included as control compounds. The solubility of nicardipine is pH dependent whereas the solubility of pyrene is pH independent.

##### Data Analysis

The solubility was estimated from the concentration of the test compound that produced an increase in absorbance above the vehicle control (i.e., 1% DMSO in buffer).

An estimated precipitation range (lower and upper bound) and a calculated mid-range value were returned, along with any relevant comments, in the form of an Excel spreadsheet.

#### 3.8.2. Microsomal Metabolic Stability

The test compound (3 μM) was incubated with pooled liver microsomes at 5 time points over the course of a 45 min experiment, and the test compound was analysed by LC-MS/MS. Intrinsic clearance values (CL_int_) with standard error and t_1⁄2_ values were measured.

A total of 50 μL of the 10 mM test compound in DMSO per species per assay condition.

##### Experimental Procedure

Microsomes (final protein concentration 0.5 mg/mL), 0.1 M phosphate buffer pH 7.4, and the test compound (final substrate concentration 3 μM; final DMSO concentration 0.25%) were pre-incubated at 37 °C prior to the addition of NADPH (final concentration 1 mM) to initiate the reaction. The final incubation volume was 50 μL. A minus cofactor control incubation was included for each compound tested where 0.1 M phosphate buffer pH 7.4 was added instead of NADPH (minus NADPH). Two control compounds were included with each species. All incubations were performed singularly for each test compound.

Each compound was incubated for 0, 5, 15, 30, and 45 min. The control (minus NADPH) was incubated for 45 min only. The reactions were stopped by transferring 20 μL of incubate to 60 μL methanol at the appropriate time points. The termination plates were centrifuged at 2500 rpm for 20 min at 4 °C to precipitate the protein.

##### Quantitative Analysis

Following protein precipitation, the sample supernatants were combined in cassettes of up to 4 compounds, the internal standard was added, and samples were analysed using Cyprotex generic LC-MS/MS conditions.

##### Data Analysis

From a plot of ln peak area ratio (compound peak area/internal standard peak area) against time, the gradient of the line was determined. Subsequently, half-life and intrinsic clearance were calculated using the equations below:

elimination rate constant (k) = (- gradient)(1)

half-life (t_1⁄2_)(min) = 0.693/k (2)

intrinsic clearance (CL_int_)(μL/min/mg protein) = V × 0.693/t_1⁄2_ (3)

V = Incubation volume (μL)/Microsomal protein (mg)(4)

relevant control compounds were assessed, ensuring intrinsic clearance values fall within the specified limits.

#### 3.8.3. In Vitro Cardiotoxicity; hERG Channel Inhibition (IC_50_ Determination)

Mammalian cells expressing the hERG potassium channel were dispensed into 384-well planar arrays and hERG tail-currents measured by whole-cell voltage-clamping. A range of concentrations of the test compound was then added to the cells and a second recording of the hERG current was made. The percent change in hERG current was calculated and used to calculate an IC_50_ value (test compound concentration which produces 50% inhibition). IC_50_ is delivered, standard error (SE IC_50_), and n (number of data points used to calculate IC_50_) for the test compound. The experiments were performed on an Ion Works TM automated patch clamp instrument (Molecular Devices LLC (San Jose, California, United States)), which simultaneously performs electrophysiology measurements for 48 single cells in a specialised 384-well plate (PatchPlateTM(manufacturer, city, state if USA or Canada, country)). All cell suspensions, buffers, and test compound solutions were at room temperature during the experiment.

The cells used were Chinese hamster ovary (CHO) cells stably transfected with hERG (cell-line obtained from Cytomyx, UK). A single-cell suspension was prepared in extracellular solution (Dulbecco’s phosphate buffered saline with calcium and magnesium pH 7.2) and aliquots added automatically to each well of a Patch Plate TM. The cells were then positioned over a small hole at the bottom of each well by applying a vacuum beneath the plate to form an electrical seal. The vacuum was applied through a single compartment common to all wells which was filled with intracellular solution (buffered to pH 7.2 with HEPES). The resistance of each seal was measured via a common ground-electrode in the intracellular compartment and individual electrodes placed into each of the upper wells.

Electrical access to the cell was then achieved by circulating a perforating agent, amphotericin B, underneath the PatchPlateTM. The pre-compound hERG current was then measured. An electrode was positioned in the extracellular compartment and a holding potential of −80 mV applied for 15 s. The hERG channels were then activated by applying a depolarising step to +40 mV for 5 s and then clamped at −50 mV for 4 s to elicit the hERG tail current, before returning to −80 mV for 0.3 s.

Compound dilutions were prepared by diluting a DMSO solution (default 10 mM) of the test compound using a factor 5 dilution scheme into DMSO, followed by dilution into extracellular buffer such that the final concentrations tested were typically 0.008, 0.04, 0.2, 1, 5, and 25 μM (final DMSO concentration 0.25%). The Ion Works TM instrument automatically adds test compound dilutions to the upper wells of the PatchPlateTM. The test compound was left in contact with the cells for 300 s before recording currents using the same voltage-step protocol as in the pre-compound scan. Quinidine, an established hERG inhibitor, was included as a positive control and vehicle control (0.25% DMSO) as negative control.

Each concentration was tested in 4 replicate wells on the PatchPlateTM (maximum of 24 data points). Filters were applied to ensure only acceptable cells were used to assess hERG inhibition. The cell must maintain a seal resistance of greater than 50 MOhm and a pre-compound current of at least 0.1 nA and ensure cell stability between pre-and post-compound measurements.

For each replicate, the hERG response was calculated using the following equation:

% hERG response = [Post-compound current (nA)/Pre-compound current (nA)] × 100(5)

The % hERG response was plotted against concentration for the test compound and, where concentration-dependent inhibition was observed, the data were fitted to the following equation and an IC_50_ value calculated:

y = [y_max_ − y_min_/1 + (IC_50_/x)^s^]+ y _min_ (6)

where y = hERG response; y_max_ = mean vehicle control response; x = concentration; IC_50_ = concentration required to inhibit current by 50%; s = Hill slope.

## 4. Conclusions

So far, the main approach for FAK inhibition has been focused on FAK catalytic function, which mainly targets the Y397 tyrosine phosphorylation site by interfering with the binding of ATP. This has resulted in the development of several FAK kinase inhibitors such as defactinib in the clinical stage. However, this approach lacks specificity with varying degrees of cross-reactivity with other tyrosine kinases and associated unwanted side effects. Alternatively, the inhibition of the FAK scaffolding function and the downstream signalling is believed to be a favourable strategy, and likely offers more specific FAK inhibitors rather than focusing solely on the kinase function. In this study, we used a structure based virtual screening approach to identify a small molecule hit compound **I**. Subsequently, a series of compound **I** analogues was synthesised and screened for antiproliferative activity in three breast cancer (MDA-MB-231, T47D, BT474) cell lines and one pancreatic cancer (MIAPaCa2) cell line. Compounds I and **5f** displayed low micromolar antiproliferative activity across the four cell lines. Moreover, compounds I and **5f** have promising metabolic stability in the liver microsome, and compound 5f lacks cardiotoxicity in the hERG inhibition assay. With no FAK inhibitors currently on the market, compound **5f** represents a promising drug-like small molecule that justifies further development as a lead compound for breast and pancreatic cancer therapy.

## Figures and Tables

**Table 1:** In vitro anti-proliferative activity (IC_50_ in μM) of compounds (**I**, **5a**–**i**) compared to chloropyramine (**C4**) in human breast (MDA-MB-231, T47D, and BT474) and pancreatic (MIAPaCa2) cancer cell lines. IC_50_ values represent the mean of independent triplicate experiments.

ID	R_1_	R_2_	MDA-MB-231 ER^−^ PR^−^ HER^−^	T47D ER^+^ PR^+/−^ HER^−^	BT474 ER^+^ PR^+/−^ HER^+^	MIAPaCa2
**I**	4-^t^Bu	4-Cl	6.37	6.71	2.67	5.66
**5a**	4-^t^But	4-OCF_3_	5.63	4.94	5.00	5.51
**5b**	4-^t^But	4-SCF_3_	11.78	11.44	12.46	15.72
**5c**	4-^t^But	4-F	12.76	13.38	8.94	15.12
**5d**	4-^t^But	2-F	14.79	14.74	14.78	15.80
**5e**	4-^t^But	3-F	14.12	14.38	14.97	16.97
**5f**	4-^t^But	4-^t^But	5.06	4.59	4.70	5.28
**5g**	4-OCF_3_	4-Cl	16.95	15.26	15.53	45.32
**5h**	4-OCF_3_	4-OCF_3_	12.75	13.23	13.19	39.89
**5i**	4-OMe	4-OMe	46.25	43.93	44.50	49.04
**C4**	-	-	99.02	>100	>100	>100

**Table 2:** In vitro aqueous solubility of diarylethylamine salt analogues (**5a**–**5c, 5f,** and **5h**) compared to nicardipine and pyrene (as control compounds) in buffered aqueous solution.

ID	Estimated Precipitation Range (µM)		
	Lower Bound	Upper Bound	Calculated Mid-Range
**Compound I**	100	>100	>100
**5a**	30	100	65
**5b**	3	20	11.5
**5c**	100	>100	>100
**5f**	1	6.5	3.75
**5h**	30	100	65
**nicardipine**	10	30	10.5
**pyrene**	3	10	6.5

**Table 3:** In vitro metabolic stability of compounds **5a, 5c, 5f**–**h** compared to control compounds (dextromethorphan and verapamil) in human liver microsomes; CL_int_ = intrinsic clearance, SE CL_int_ = standard error, t_1/2_ = half-life, and n = number of time points used to calculate CL_int_.

ID	Metabolic Stability (Human)									
	CL_int_ (μL/min/mg protein)	SE CL_int_	t_1/2_ min	n	Compound Remaining (% of 0 min)					
					0 min	5 min	15 min	30 min	45 min	Control
**Compound I**	9.10	3.35	152	5	100	89.4	92.3	77.9	81.3	95.7
**5a**	8.91	0.532	156	5	100	95.8	92.3	86.0	81.4	91.0
**5c**	8.26	1.15	168	5	100	94.7	95.0	86.1	82.5	98.0
**5f**	9.34	0.699	148	5	100	97.2	92.2	88.0	80.7	88.0
**5g**	13.9	2.14	99.9	5	100	99.0	96.2	86.8	72.8	86.6
**5h**	2.35	2.31	589	5	100	102	108	100	95.8	97.8
**dextromethorphan**	28.2	3.73	49.2	5	-	-	-	-	-	-
**verapamil**	187	2.82	7.40	3	-	-	-	-	-	-

**Table 4:** In vitro cardiotoxicity expressed in terms of IC_50_ of hERG channel inhibition of diarylethylamine compounds (**5a**, **5f**, **5i**) compared to control compounds (quinidine and dimethylsulfoxide). SE IC_50_: standard error; n: number of data points used to calculate IC_50_.

ID	hERG Channel Inhibition		
	IC_50_ (µM)	SE IC_50_ (µM)	n
**Compound I**	5.47	0.616	15
**5a**	3.59	0.833	11
**5f**	>25	-	12
**5i**	6.01	1.22	11
**quinidine**	1.39	0.366	12
**dimethylsulfoxide**	>25	-	15

## Supplementary Materials

Supplementary data associated with this article can be found in the online version. These data include Figure S1; Western blot analysis of compound **I** effects on activation and total stability of FAK. Figures S2–S4; the spectroscopic data of compound **5f** (^1^H, ^13^C, ^19^F NMR, and mass spectrometry).
