# Supplementary material for: Structure-Based Virtual Screening, Synthesis and Biological Evaluation of Potential FAK-FAT Domain Inhibitors for Treatment of Metastatic Cancer
Source: Molecules. 2020 Jul 31;25(15):3488. doi: 10.3390/molecules25153488 (PMC7435868; doi:10.3390/molecules25153488)
Supplement: Supplementary file 1 [file molecules-25-03488-s001.pdf]

Supporting Information

Structure based virtual screening, synthesis and biological evaluation of potential FAK-FAT domain inhibitors for treatment of metastatic cancer

Sahar B. Kandil*, Samuel R. Jones, Sonia Smith, Stephen E. Hiscox and Andrew D. Westwell

School of Pharmacy and Pharmaceutical Sciences, Cardiff University, Redwood Building, King Edward VII Avenue, Cardiff CF10 3NB, Wales, UK

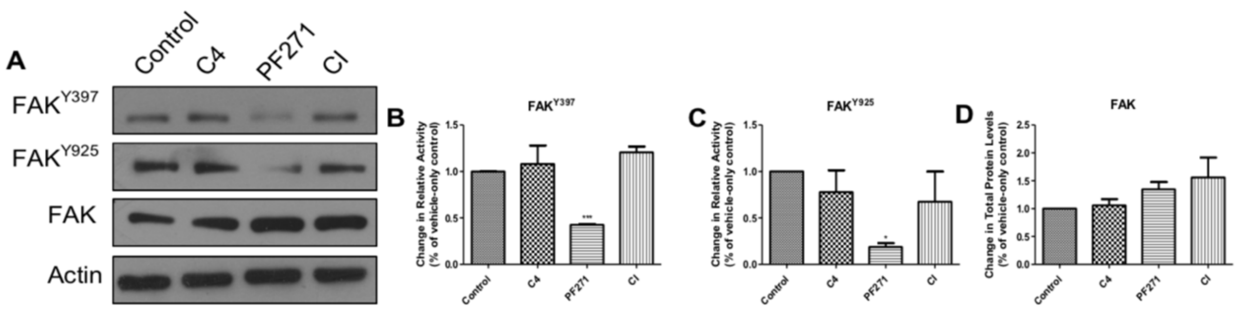

Figure S 1. Western blot analysis of the effects of compound **I (CI)** on activation and total stability of FAK **(A).** Chloropyramine (C4) and PF271 were also examined. From western blots, levels of specific protein activities were calculated using densitometry **(B-D).** All error bars are representative of SEM; n=3. *p<0.05; **p<0.01; ***p<0.001.

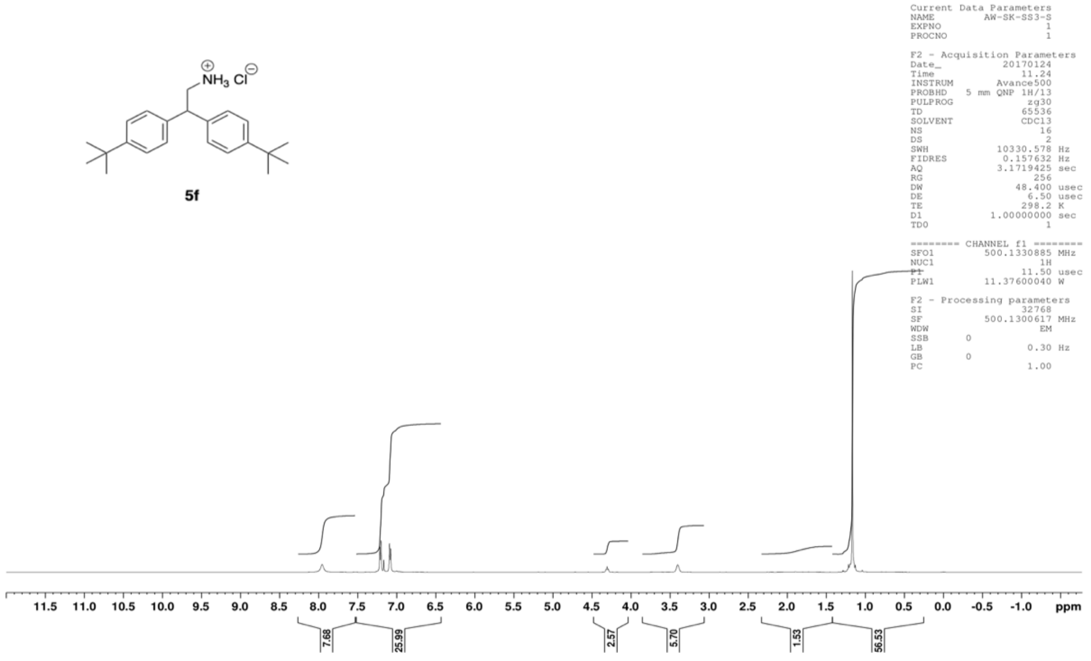

Figure S 2. ^{1}H NMR spectra of compound 5f

* Email: kandils1@cf.ac.uk

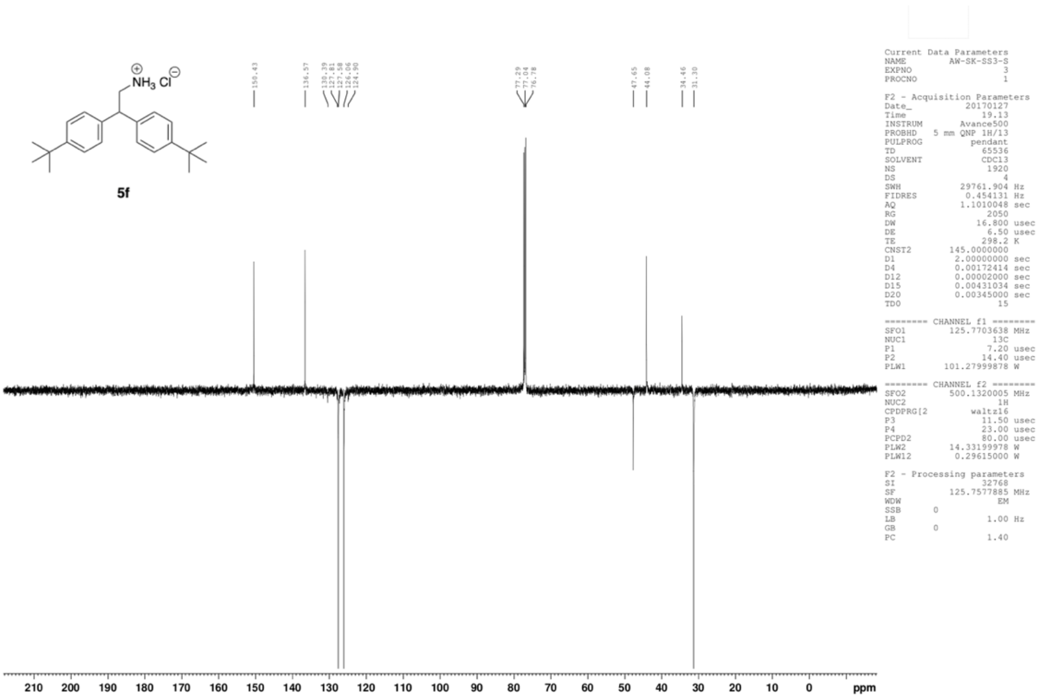

Figure S 3. ^{13}**C NMR spectra of compound 5f**

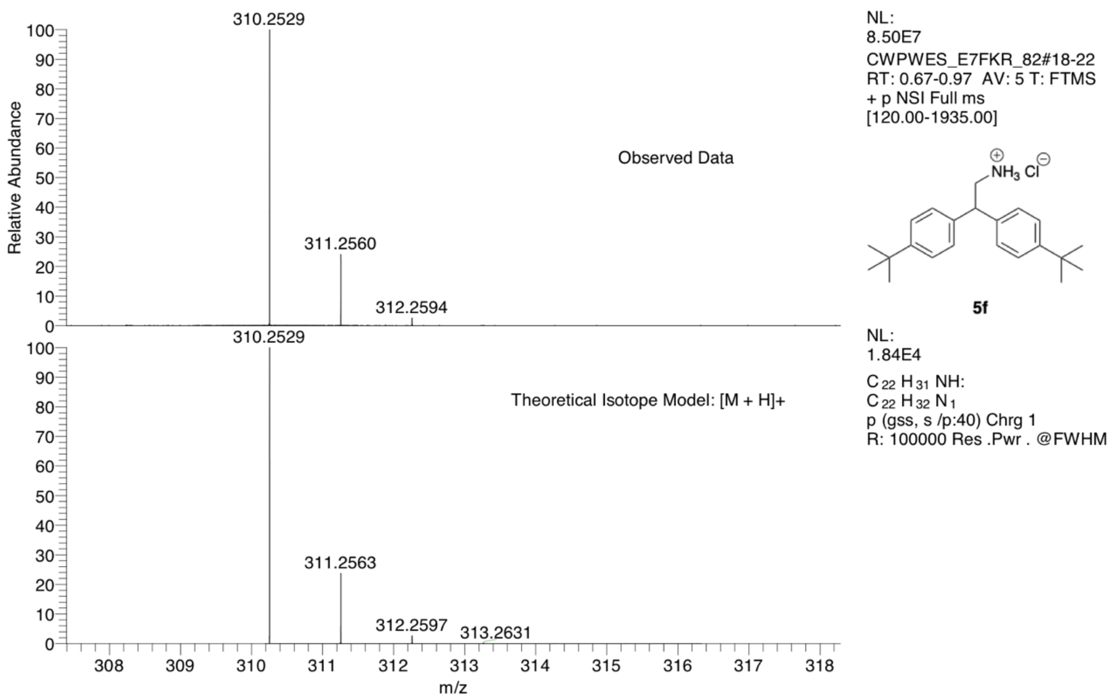

Figure S 4. Mass spectra of compound 5f
